# Establishing genome sequencing and assembly for non-model and emerging model organisms: a brief guide

**DOI:** 10.1186/s12983-025-00561-7

**Published:** 2025-04-17

**Authors:** Tilman Schell, Carola Greve, Lars Podsiadlowski

**Affiliations:** 1 LOEWE Centre for Translational Biodiversity Genomics, Senckenberganlage 25, 60325 Frankfurt, Germany; 2 Senckenberg Research Institute, Senckenberganlage 25, 60325 Frankfurt, Germany; 3 LIB, Museum Koenig Bonn, Centre for Molecular Biodiversity Research (zmb), Adenauerallee 127, 53113 Bonn, Germany

**Keywords:** De novo genome assembly, Long-read sequencing, Sequencing quality check, Assembly metrics, Genome annotation, High molecular weight DNA

## Abstract

Reference genome assemblies are the basis for comprehensive genomic analyses and comparisons. Due to declining sequencing costs and growing computational power, genome projects are now feasible in smaller labs. De novo genome sequencing for non-model or emerging model organisms requires knowledge about genome size and techniques for extracting high molecular weight DNA. Next to quality, the amount of DNA obtained from single individuals is crucial, especially, when dealing with small organisms. While long-read sequencing technologies are the methods of choice for creating high quality genome assemblies, pure short-read assemblies might bear most of the coding parts of a genome but are usually much more fragmented and do not well resolve repeat elements or structural variants. Several genome initiatives produce more and more non-model organism genomes and provide rules for standards in genome sequencing and assembly. However, sometimes the organism of choice is not part of such an initiative or does not meet its standards. Therefore, if the scientific question can be answered with a genome of low contiguity in intergenic parts, missing the high standards of chromosome scale assembly should not prevent publication. This review describes how to set up an animal genome sequencing project in the lab, how to estimate costs and resources, and how to deal with suboptimal conditions. Thus, we aim to suggest optimal strategies for genome sequencing that fulfil the needs according to specific research questions, e.g. “How are species related to each other based on whole genomes?” (phylogenomics), “How do genomes of populations within a species differ?” (population genomics), “Are differences between populations relevant for conservation?” (conservation genomics), “Which selection pressure is acting on certain genes?” (identification of genes under selection), “Did repeats expand or contract recently?” (repeat dynamics).

## Introduction

Genomics, the determination of genome sequences and their comparison within populations and between species, has transcended traditional biological boundaries, profoundly influencing various scientific disciplines, from basic questions in ecology and evolutionary biology to applied approaches in medicine and agriculture. The genome sequence, encompassing all its genes, regulatory elements, and other non-coding regions, serves as a foundation for unravelling the function of individual genes and their interactions within biological systems. Functional genome annotation involves assigning functions to encoded proteins and helps to understand the roles of genes within physiological pathways or developmental processes. Reference genomes provide the basis for large scale comparative approaches within and between species. Evolutionary dynamics can now be followed genome wide by monitoring genomic variation through time and space.

The Human Genome Project (HGP), published as a preliminary draft genome in 2001 [1, 2], marked a watershed moment in genomics. This almost complete genome sequence provided a blueprint to study human biology in an unprecedented way, paving the way for linking genetic variation, gene expression, and complex gene interactions to phenotypic traits, mainly to understand the genetic basis of human diseases [3, 4], but also providing the foundation for a broad overview of genetic variation in our species [5].

While the HGP brought important progress for several aspects of medical research, the parallel running genome projects of other multicellular organisms also allowed for more experimental approaches. Sequencing the genomes of classical model organisms, like the nematode worm *Caenorhabditis elegans* [6], the fruit fly *Drosophila melanogaster* [7], the beetle *Tribolium castaneum* [8], the mouse *Mus musculus* [9], and the flowering plant *Arabidopsis thaliana* [10]. For these model organisms there are now established genetic toolkits available, like RNAi [11, 12], CRISPR/CAS [13] or TALEN [14], which enable to silence genes and to study the effects of induced variants, and thus linking genotypes and phenotype with experimental approaches.

The rapid rise of genomic studies after these ground-breaking initiatives was strongly promoted by the advent of high-throughput sequencing technologies in the years after 2005 [15]. Large amounts of sequence information could be gained in less time and for less money. Initially, short-read technologies (producing sequence reads of 50–300 bp) ruled the market, heavily used in re-sequencing of human genomes for medical science [16] and population genomics of humans and model organisms [5, 17].

Meanwhile, long-read sequencing approaches, recently coined “method of the year” by Nature Methods [18], are the method of choice and allow for much better genome assemblies up to chromosome-scale scaffolds [19]. Comparative genomic studies are performed across all the branches of the tree of life [20–22]. Advances in sequencing technology and assembly techniques promoted large consortium efforts to produce datasets that allow for broad scale comparative genomics approaches and led to the final goal to sequence representative genomes from all eukaryotic species (www.earthbiogenome.org; [23]).

Comparative genomics enabled scientists to identify conserved genes and pathways, elucidating their fundamental roles in biological processes and their evolutionary adaptive changes [24] and has facilitated the discovery of many genes that lack orthologs in other taxa, so-called “orphan” genes [25, 26], thereby shedding light on the evolution of new genes. Based on genomic data, the complex interplay of multiple genes responsible for quantitative traits can be unravelled through genome-wide association studies (GWAS) [27]. Comparative genomics on the level of populations and closely related species help to understand genetic mechanisms underlying local adaptations and speciation processes. This knowledge aids in biodiversity conservation, e.g. by identifying endangered species, assessing genetic diversity, and devising conservation strategies crucial for preserving ecosystems [28, 29].

The surge in genomic data has propelled not only methods in laboratory analyses but also the field of bioinformatics by requiring sophisticated computational tools for the analysis of huge genomic datasets and their interpretation [30]. Public databases and genome browsers were optimised to deal with the new datasets, while machine learning algorithms, data mining techniques, and high-performance computing have become indispensable in deciphering complex genomic information, taking biological research to a higher level [31].

Numerous reviews are available to accompany a newly envisioned genome project, e.g. Dominguez del Angel et al. 2018 [32] provided general considerations divided into ten important steps for genome sequencing, assembly and annotation. Kim and Kim [33], provide a step by step workflow example with scripts to assemble a *Drosophila* genome and detect structural variants; Li and Durbin [34] provide an overview on assembly strategies for chromosome-level approaches; on the analytical part Lariviere et al. [35] provide access to Galaxy workflows for the analysis of vertebrate genomes. There are also guides for genome projects in population and conservation genomics [36–38].

**Fig. 1:**
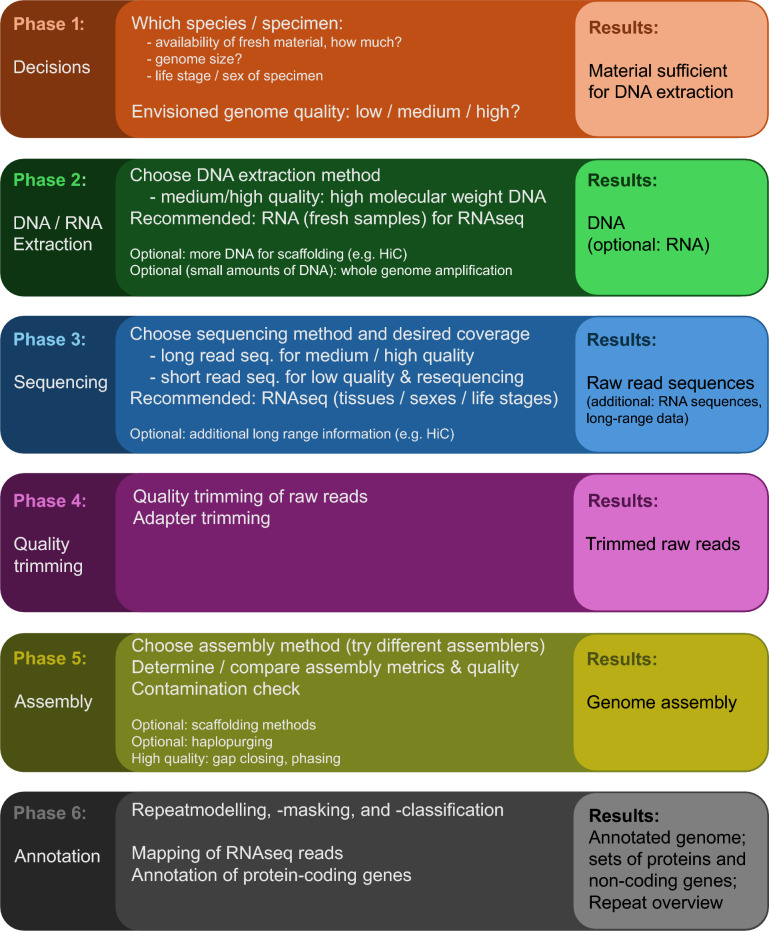
Schematic overview about different phases of prior considerations (phase 1), laboratory procedures (phase 2 and 3), and analytical steps (phase 4–6) to generate a de-novo assembly and annotations

As the options offered by different sequencing techniques are rapidly evolving, and the common standards are moving towards perfection, this review aims to provide up-to-date advice on starting a new genome project for emerging model organisms with general considerations on the final assembly quality adapted to different research questions, and which techniques to combine for an optimal and cost-effective approach. In this review we present a guide for the different steps of a genome project (Fig. 1), starting with database mining, a cost estimate and sample selection (phase 1), the wet lab steps of DNA extraction, optional whole genome amplification (phase 2) and sequencing (phase 3), followed by analytical steps with the sequencing data, like quality trimming (phase 4), assembly plus quality checks (phase 5) and briefly also address annotation of repeats and genes (phase 6).

## Which questions can be answered by which kind of genomic analysis

Short read-based genome sequencing with low to medium coverage (5× − 20× sequence data in comparison to genome size) might be useful when a reference genome is already available for that species and a population genomics study is envisioned, e.g. for conservation issues or to identify genes under selective pressure; but note that some reference genomes are highly fragmented and might lack information about gene location (annotation). Phylogenomic datasets of single copy orthologs can be generated from low coverage genome assemblies. In general, pure short-read data is not recommended for generating a new reference genome. However, this may be the only option if the extraction of good quality DNA (= containing a substantial fraction of large fragments) is not possible, e.g. from museum collection material. For taxa with smaller genomes, precious samples such as holotypes [39, 40] or simply in projects with limited financial resources short-read assemblies can provide useful genome assemblies, that can be used e.g. for SNP comparison in population genomics approaches, as well as for the comparative analysis of nuclear markers and for the design of PCR primers and baits for hybrid enrichment follow-up studies [41–43].

Chromosome-level genome projects are now the common goal of most community driven reference genome projects. Usually done with long-read sequencing, resulting contigs will most often be accompanied with additional scaffolding information (e.g. from Hi-C data, see phase 3). The resulting scaffolds still might have a substantial part of undetermined sequence, and several contigs may remain not assigned to any chromosomes. Thus, chromosome level assemblies are not 100% complete. Anyway, they enable comparative genomics studies focusing on genome structure, selection and gene family evolution with respect to ecology of the species. Furthermore, chromosome-scale assemblies allow for the reconstruction of ancestral linkage groups and help to understand patterns and reasons for genome size variation in closely related groups. E.g. expansion of repeat elements was shown to be the main reason for genome size increase in the Wood-White butterflies [44] or caddisflies [45].

The highest quality of genome assemblies are termed telomere-to-telomere (T2T) assemblies [46], referring to the complete gapless sequence information from one end of the chromosome to the other (including centromeres), while the telomeres (the tips of the chromosomes) themselves are only partly covered, as they tend to be composed of simple repeats, which lengths are variable even between cells of the same organism [47, 48]. For the human genome project, although officially finished in 2003, it was estimated to lack about 7% of the sequence [49]. A complete, gap-free sequence was not available before 2022 [50], still lacking the repeat-rich complete sequence of the Y chromosome, which followed in 2023 [19]. Thus, most of the published genome sequences of eukaryotes can be interpreted to be in different stages of incompleteness. T2T sequencing allows for the recognition of otherwise hidden structural dynamics of genome evolution. E.g. T2T sequences of 142 strains of yeast genomes revealed more than 4000 structural variants including large deletions and translocations as well as regions acquired by horizontal transfer [51]. However, telomeres and centromeres still provide sequencing challenges due to low complexity and content of simple repeats [52–54]. As well male sex chromosomes, at least in mammals, are difficult to assemble due to their degeneration through gene loss and accumulation of repeats and palindrome sequences [55]. In some species mini- or micro-chromosomes are part of the genome, often difficult to identify among the genomic contigs [56]. Songbirds on the other hand are an example for species with germline restricted chromosomes [57], which will be without sequence information, when only somatic tissue is used for DNA extraction.

## Overview of the steps for a genome project

Here we give a brief overview and introduce some of the terminology, with more details following in later chapters (Fig. 1).

Phase 1: Decisions. The first step is to get an overview about available genomes in public databases and ongoing large consortia genome projects. Depending on the budget it must be decided if one or more genomes will be part of the project (e.g. many individuals in a population genomics project or several species for comparative genomics or phylogenomics). Amount and quality of the samples might be a point here to select which specimens and/or species will be included. As well a decision about the envisioned assembly quality (low coverage, chromosome level, T2 T) should be done early in the process, although genome projects can be “upgraded”, e.g. by generating more data later, given that sufficient sample material is left for that purpose. Knowledge about the genome size is very useful before starting a genome project, because together with the envisioned sequencing depth it defines the amount of sequence data to be generated and therefore is crucial for assessing the cost of the project.

Phase 2: DNA extraction: The first wet-lab part in a genome project is the extraction of DNA from the organisms under study. Ideally, high-molecular weight (HMW) DNA in a sufficient amount can be generated. HMW means that most of the DNA fragments have lengths of > 10 kbp, at best much longer. Besides the sample quality (the fresher, the better) also the DNA isolation approach has a strong influence on the DNA fragmentation. While classical DNA extraction protocols tend to fragment DNA unnecessarily, a couple of special methods to yield HMW DNA will be suggested here. In cases where sufficient DNA amount is lacking, there are methods to amplify DNA randomly or specific low-input protocols can be used. These methods are discussed in a separate chapter.

Phase 3: Library preparation and sequencing. Many researchers will just send HMW DNA to a sequencing company and get back the raw sequencing data. However, a better knowledge of the library preparation and sequencing steps may help to understand which the best way is to assemble the genome. For those who are actively sequencing in the lab, we provide an overview about library preparation and sequencing of the two most popular long-read sequencing approaches, Oxford Nanopore (ONT) and PacBio HiFi.

Phase 4: Quality trimming. After sequencing, several quality checks and trimming steps will be performed on the raw sequence data. Sequence read quality is assessed during the sequencing procedure and is encoded together with the pure sequence. Parts of the sequence that have bad quality (= are less reliable, error-prone) can be filtered from the sequence reads. Besides this quality trimming, adapter trimming is done because most sequencing methods use adapters (extra chunks of DNA of known base sequence that are ligated to the DNA fragments of interest to prepare them for NGS sequencing) that may be accidentally sequenced together with the desired sequences.

Phase 5: Assembly. Prepared in that way, the sequenced fragments can be used for an assembly procedure, which will use overlaps between sequence reads to create longer contiguous sequence, the so-called “contigs”. Additional steps are also helping to get better assemblies, forming scaffolds (= contigs linked by additional evidence, often including gaps of defined size, which are displayed as multiple Ns in the sequence). One prominent method used heavily in recent genome projects is Hi-C, which exploits the physical neighbourhood of regions in condensed chromosomes to provide linkage information within a chromosome, allowing for chromosome-scale assemblies. The final assemblies of a genome project should be subject to quality checks, especially when several assembly approaches will be compared. Here a filtering step for foreign contamination is useful to identify sequences from laboratory and natural contaminants (e.g. symbiotic bacteria).

Phase 6: Annotation. Comparison of the assembled genome with reference genomes from closely related (or the same) species is often part of downstream analyses after assembly. However, often a de novo annotation of repeats and genes is necessary. Annotation is not the main focus of this review, but we give some advice for the first steps here. Currently, this field is heavily affected by new methods from the field of machine learning using e.g. protein language models.

## Decisions before starting the project (phase 1)

### Data mining

Before starting a genome project, it is recommended to browse databases to find out about available genome data and ongoing initiatives, as the envisioned genome project may already be underway in another laboratory. There are many genome initiatives around the world producing new genome records with growing speed. Besides looking up the genome records in NCBI datasets (https://www.ncbi.nlm.nih.gov/datasets) there are numerous individual listings on the websites of e.g. the earth biogenome project (https://www.earthbiogenome.org) [23], Darwin tree of life, focusing on the British fauna and flora (https://darwintreeoflife.org) [22], Genome 10 k, aiming for 10.000 vertebrate genomes (https://genome10k.ucsc.edu), and i5k, focusing on arthropod diversity (https://i5k.github.io). A good starting point is the *Genomes on a tree* hub (https://goat.genomehubs.org), which aims to provide an overview of completed and ongoing genome projects using a phylogenetic approach (e.g. getting all entries for a higher ranked taxon with associated information such as assembly size and chromosome numbers) [58].

### Determining genome size and ploidy level

There are several key metrics that should ideally be available before starting a genome sequencing project, predominantly genome size and ploidy level (= how many homologous chromosome sets are usually present in a cell of that organism). A reliable genome size estimate is needed to calculate the amount of data to be generated during sequencing. Extremely large genome sizes can be a reason to cancel a project before it starts, if not enough resources can be spent to generate sufficient sequence data. A lot of resources would be wasted, if data had already been generated and during the assembly process the concern arises that the genome is too big for an accurate assembly with the generated data. Furthermore, comparing the estimated genome size with the size of a genome assembly is an important quality criterion for completeness. Prior information on genome size from many published studies is available in the animal genome size database [59] and in “genomes on a tree” [58]. If the species is not in the database a look at close relatives may help, but is not always reliable, as genome expansion or reduction can occur even within families and genera [45, 60].

If no reliable information is available, genome size can be determined. There are two main ways to conduct measurements: sequence free methods and approaches relying on existing short-read sequencing data. By far the most common sequence free method used today is flow cytometry [61]. This method compares the fluorescence of stained nuclei from the sample and a standard of known genome size (e.g. chicken nuclei or cricket) in a steady flow. However, it relies on the availability of fresh or frozen tissue samples/cells and access to a flow cytometer. For proper calibration in flow cytometry, genomes of known size must be provided that are of similar (or at least not too different) genome size than the sample under study to properly calculate the unknown genome size. Knowledge about unusual ploidy level is also helpful here [62, 63].

Alternatively it is possible to estimate genome size bioinformatically from sequencing data, e.g. by k-mer based or and mapping based approaches. For k-mer based genome size estimation the k-mers (20–120 bp sequence snippets, generated from raw reads to reduce computational complexity) should be very accurate (e.g. from Illumina short reads). Firstly a histogram containing the k-mer profile is created, e.g. with jellyfish [64]. The resulting k-mer distribution can be modelled, e.g. with GenomeScope [65], to filter out sequencing errors, infer genome size, heterozygosity, and repeat content. Genome size is in principle determined by dividing the total available sequence amount by the value of the peak of the k-mer frequency density plot. Multiple peaks may also be a hint to massive contaminations, e.g. by symbiotic microorganisms. While k-mer based genome size estimates are commonly used, mapping based estimates such as ModEst [66] can be more accurate.

Most animals have diploid genomes. However, some show haploid tissues (e.g. male hymenoptera), others have polyploid genomes, as many plant species, and animals like *Xenopus* frogs [67], or various fishes [68], often following a history of hybridisation events. This may have consequences for assembly and annotation. Ploidy level might also be variable across tissues [69]. Especially when SNP calling in resequencing projects/population genomics projects is desired, ploidy level of the species should be known. It can be determined by karyotyping. However, karyotyping (= preparation of metaphase chromosome and staining) is a tedious process that requires lots of experience and living tissue [70]. It is therefore understandable that new projects may not be able to obtain information on a yet unknown karyotype prior to sequencing.

### Envisioned genome quality and sequencing approach

The next consideration is about which quality of the genome assembly is aimed for, because this also affects the costs of the project (see below). Ideally a complete genome assembly should contain all the nucleotides of each chromosome in a contiguous sequence. In practice this is rarely achieved. Many genome projects in the past delivered genome assemblies as a set of (often thousands of) contigs or (hundreds of) scaffolds. Genome assemblies are referred to as “chromosome-level” when these scaffolds are close to the size of chromosomes (or chromosome parts in case of metacentric or submetacentric centromeres which are often not well covered in assemblies due to their repeat structure). Long-read sequencing is the core of modern genome sequencing. Whether PacBio or Oxford Nanopore (ONT) is the better choice is difficult to decide. Output of the newest generations of machines is similar, read quality is best with PacBio HiFi (error rate below 0.1%), but read length is usually not higher than 15 kb. ONT can generate a fraction of longer reads (some > 100 kb) but has still an error rate of 1–2% in the latest generation of flowcells. As a true single-molecule sequencer only ONT offers the possibility to detect base modifications directly in the reads (e.g. methylation). On the other hand, PacBio has a couple of low input protocols for smaller amount of DNA (see below). If affordable, a combination of both methods will probably yield better assemblies than any of the single approaches alone.

Sequence information of the two homologous chromosomes may be mixed within a genome assembly (e.g. one sequence representing variants from both haplotypes). For some research questions it is desired to separate the haplotype variants of the two chromosome sets, which is referred to as phasing. The combination of long reads and Hi-C reads can help to separate both haplotypes of a diploid organism. If parents and offspring were sampled, short-read sequencing data from both parents might also help here. Phasing creates a more accurate representation of the genome compared to non-phased assemblies, which contain both haplotypes mixed in the same sequence (e.g. primary contigs). In the case of a phased assembly, there will be two separate assemblies available, usually one of them containing the autosomes and debris of haplotype A, the sex chromosomes and the mitochondrial genome and the other one containing the autosomes and debris of haplotype B. While phased assemblies are of higher quality compared to non-phased assemblies, e.g. comparative and population genomic analyses are still possible.

Recent genome initiatives, for example under the umbrella of the Earth BioGenome Project (https://www.earthbiogenome.org), have set standards to be fulfilled in genomic sequencing and assembly [71]. Often the aim is to reach the best possible level of contiguity and completeness, at best a chromosome-scale or telomere-to-telomere assembly [34, 72]. Raw data requirements include sufficient coverage (at least 30×) with long reads [73] and additional sequence data for additional scaffolding steps (e.g. 50× coverage with Hi-C data) [71]. To provide genome information that is useful for future researchers it is recommended to aim for similar standards in “private” genome projects as well.

### Sample selection

To reduce genetic variation, it is generally preferable to use only a single individual for genome sequencing. If it is unavoidable to use more than one individual, biological differences should be minimized, e.g. by using clones (for example *Daphnia*), individuals from one breed/strain of a lab culture or even from the same wild population (a second individual for long-range information, e.g. Hi-C sequencing, a third individual for RNA sequencing, which aids in gene annotation (here maybe several RNA samples to represent different sexes and tissues, life stages). Depending on the sex determination mechanism in the species, it may also be necessary to consider which sex to select. The heterogametic sex will represent all chromosome types, while the sex chromosomes will only have half coverage compared to the other chromosomes (autosomes) in the resulting genome assembly, which can cause problems in assembly and in subsequent downstream analyses due to the partially duplicated nature of the sex chromosomes [74]. As well, the mammalian sex chromosome Y presents a challenge for assembly due to its high repeat content [75].

If the organisms are not too small, the choice of tissue for high molecular weight (HMW) DNA extraction is of importance: to avoid subsequent contamination, it is advisable to choose tissue without intestinal tract (contamination with food or bacteria; digestive enzymes may also damage DNA) or tissue with as little potential of contamination as possible (e.g. for vertebrates: brain, spleen, kidney, liver, muscle, blood; not recommended is the use of tissues with a high fat content or vertebrate bone). When flash frozen tissue was selected, tissue type had no significant effect on DNA fragment length, with blood samples tending to provide the highest and least degraded DNA yields in vertebrates [76] while being basically free of contamination.

If phasing (separate assemblies of each chromosome haplotype) is desired it has been recommended in the past to sample trios of parents and offspring, which helps to distinguish individual chromosomes from each parent [77]. Besides this approach phasing can also be done with accurate long-read data without parental information [78, 79] or long-range information like Hi-C [80].

### Estimating costs and bioinformatic resources

How much sequencing data is needed for a successful genome project has to be calculated from genome size and desired coverage. If a reference genome is already available, about 20 × coverage will allow to recover most of the variants and heterozygous sites (single-nucleotide polymorphisms, SNPs), while single copy orthologs for phylogenomic datasets might also be sufficiently found with less coverage (5x− 10x). In both cases, short reads, e.g. from an Illumina platform will give good results; we will not discuss these re-sequencing approaches in more detail here. For a de novo genome assembly usually 30–50× with long reads (e.g. from Oxford Nanopore or PacBio platforms) should give a good representation of all parts of a genome and a useful initial assembly.

There is always sequencing data that will be filtered out before analysis (bad quality, adapter contamination, foreign contamination, reads too short). So, when the desired assembly coverage is 30x, probably 10–25% more initial sequencing data has to be produced. Anyway, the success of long-read sequencing is a bit unpredictable, so often a second round of sequencing must be done to fulfil all needs. Additional expenses has to be calculated when considering additional Hi-C data for scaffolding and RNAseq data to support the annotation process. Exact prices vary too much between companies and from year to year, so that we cannot provide reliable information here. As a rough estimate, a small sized genome (200–300 Mb) for a de novo sequencing approach in 2024 required around 500–1000 € consumable costs, if a long-read sequencer is at hand, while companies are likely to will take more than 1000 € for a single genome. While a mammalian genome (3 Gb) can be sequenced for roughly 1000 € in the lab, costs from companies including Illumina data from a Hi-C library and some RNAseq data may sum up to 2000–3000 €. Consumable costs for long-read sequencing in the laboratory is lower when many genomes need to be sequenced, as the price of consumable in bulk is often dramatically lower per unit than for single experiments.

Bioinformatic resources required for genome assembly depend mainly on the genome size and desired coverage, but to some part also on the complexity of the repeat content. For a genome size up to 0.5 Gbp a Linux/Mac system with 8–16 cores and 32–48 Gb RAM may be sufficient to generate assemblies in a few hours or one day [32]. For e.g. mammalian genomes (3 Gbp) 48 cores and > 100 Gb RAM may take one or a few days depending on the sequencing depth [81]. Be aware that overlap mappings and assembly graphs (a data structure that is generated during the assembly process) use a lot of disc space, so there should be at least 10 times as much free disc space provided than there is sequencing raw data. If no servers are provided from the research institute, cloud computing is an option here, but cost prediction is not easy here, because computation power and time is not directly proportional to data amount (see above).

## DNA extraction and optional whole genome amplification (phase 2)

### DNA extraction

For a sufficient long-read sequencing approach 100 ng DNA/Gbp genome size are proposed to be sufficient by the guidelines of the earth biogenome project [71]. Fresh or flash-frozen (−80 °C) tissue or blood samples are generally preferable for HMW DNA extraction. However, it is possible to isolate good quality DNA from tissue preserved in ethanol or RNAlater (Qiagen), even at room temperature (but better kept cool when stored for longer than a few days). However, although long fragments can be extracted from RNAlater-preserved tissue, sequencing success is often lower than that gained from fresh samples (own observation). In general, DNA extraction from dry collection material [82] or formalin-fixed tissue [83] is possible, but is recommended only for short read sequencing, as the DNA is highly fragmented.

Due to the strong impact of medical science in the development of lab methods, many of the kits and protocols are optimized for human or mammalian tissue. However, many other animals (as well as plants) pose extra challenges due to compounds that interact with DNA or with the enzymes provided within the kits [84, 85]. There is no one-for-all recipe as different taxa present different types of challenges. A growing number of recipes for the isolation of HMW DNA from various organisms and tissues can be found online (https://www.protocols.io/workspaces/high-molecular-weight-dna-extraction-from-all-kingdoms).

While a simple phenol–chloroform precipitation (PCI) does a good job with many samples, phenol and chloroform are hazardous compounds that might be avoided for daily work. The use of kits specialized for HMW DNA isolation, often combined with steps to eliminate short fragments, improve sequencing output and assembly success. Among the recommendable commercial kits are MagAttract HMW DNA kit (Qiagen), ZYMO HMW (Zymo Research), Monarch HMW DNA extraction kit (NEB), innuPREP SE HMW DNA kit (InnuScreen IST), Nanobind (PacBio), among others. Cetyltrimethylammonium bromide (CTAB) methods are used often for plant, fungal, and mollusc samples to get rid of e.g. heavy loads of secondary metabolites [86].

For ONT long-read sequencing an enrichment for long fragments shows to increase yield, as short fragments will be preferred by the pores. It can be done after DNA extraction, here notable approaches involve the short read eliminator (SRE) kits (Pacbio, formerly Circulomics), which are based on salting out methods, magnetic beads (e.g. AMPure) or using the BluePippin (SAGE) machine (in principle a preparative gel electrophoresis). A cost effective method is also to generate buffers for precipitation of HMW DNA fragments on your own [87].

### Long-range PCR/whole genome amplification and ultra-low input protocols

Several long-range PCR/whole genome amplification (WGA) methods have been developed to amplify the entire genome from minute amounts of DNA. These methods are particularly useful in situations where the amount of DNA is limited, due to the small size of individuals or when aiming for single-cell genomics. This application is also often helpful for species that cannot be sequenced because contamination with secondary metabolites is precipitated with the DNA extraction or sticks to the DNA, inhibiting the subsequent sequencing reaction or clogging the pores of the ONT flow cells. The amplification step produces "clean" synthetic DNA, which can then be sequenced.

Each of these methods has its advantages and limitations, including issues related to amplification bias, error rates, uniformity of amplification, and coverage. Amplification bias means that certain regions of the genome may be amplified less than others, so that the final library is less complex and sequence coverage may vary more than usual across the genome. This can also reduce the number of heterozygous sites detected. Furthermore, modifications of the original DNA (e.g. methylation) will be lost.

Here are some common methods for long-range PCR/whole genome amplification:

PacBio's Ultra-Low Input DNA workflow generates data volumes comparable to standard input libraries from only 5 ng total HMW DNA. A PCR mix of two different polymerases is used to minimize PCR bias. This approach has been successfully used to generate high-quality genomes from individual small animals such as mosquitoes [88] and springtails [89]. However, the PCR-based amplification step reduces the insert size of the final library to 10 kb and the protocol is only recommended for genome sizes up to 500 Mb (manufacturers guidelines). A modified PacBio Ultra-Low Input protocol with an alternative polymerase (KOD Xtreme™ Hot Start DNA Polymerase, Merck), now commercially available as PacBio Ampli-Fi kit, was able to further reduce issues related to long-read sequencing and PCR bias and also exceed the previous limit of 500 Mb genome size of the previous PacBio Ultra-Low Input protocol up to 3 Gb [90, 91]. Furthermore, even this workflow with ultra-low DNA input is not yet applicable for very small animals (e.g. < 1 mm total length) or single cells.

While this is based on standard PCR techniques, Multiple Displacement Amplification (MDA) can be achieved with an isothermal reaction setup [92]. MDA (e.g. Repli-G, Qiagen) is a popular isothermal amplification method that utilizes the phi29 DNA polymerase with high processivity and strand displacement activity. It amplifies DNA by initiating random hexamer priming at multiple sites across the genome. In contrast to PCR-based WGA approaches, MDA has the advantage of producing highly accurate fragments with an average fragment length > 10 kbp. This method is also known for its ability to produce low amplification bias and is widely used e.g. for single-cell sequencing or small amounts of degraded tissue in clinical samples [93]. The usefulness of that approach was demonstrated by sequencing the genome of a microscopic invertebrate, the gastrotrich *Lepidodermella squamata* [94]. Here MDA treatment showed a uniform coverage of the genome with sequencing reads. However, MDA has its own challenges, one of the biggest being the generation of chimeric sequences [95] and there are sometimes problems with direct sequencing of these products. Biezuner et al. compared different WGA for efficiency and error rate in single-cell approaches [96].

## Sequencing (phase 3)

### Oxford nanopore long-read sequencing

The Oxford Nanopore Technologies (ONT) sequencing approach works by passing DNA molecules through nanoscale pores embedded in a membrane [97]. As the DNA passes through these nanopores, it causes characteristic disruptions in ion flow through the pore (electrical currents), which are then decoded into DNA sequences. This real-time, single-molecule sequencing technique allows for the direct reading of DNA strands, irrespective of their length. This provides an advantage over classical polymerase-based sequencing technologies that mainly rely on incorporation of fluorescently labelled nucleotides [15]. The incorporation itself and amplification steps to enhance the signal is prone to polymerase-based errors. The read length of ONT sequence reads may span > 10 kbp with a substantial proportion of ultralong reads spanning > 100 kbp, allowing researchers to tackle complex genomic regions, such as repetitive sequences or structural variations, with great accuracy [98]. The single-molecule approach also allows for the detection of base-modifications, e.g. CpG methylation [99, 100].

Despite its advantages, Oxford Nanopore sequencing does present challenges related to error rates and accuracy, particularly in base-calling due to the nature of the electrical signal analysis. Nevertheless, ongoing advances in the technology and bioinformatics tools are continuously improving its accuracy and reliability, with the latest generation of flow-cells providing a 1–2% random sequencing error rate [101]. Older flow-cell generations (< 10.0) suffered from higher error rates (5–10%) with a significant proportion of non-random insertion/deletion errors [102], making it necessary to correct ONT assemblies with sequence reads from more accurate short-read sequencing approaches, such as Illumina [103]. Although this is generally helpful it is problematic in repeat regions where a kind of “overcorrection” can occur, due to the majority of reads from multiple repeats [104].

ONT library preparation usually involves steps with DNA fragment size selection to enrich longer DNA fragments. The standard protocol uses AMPure beads (1:1) for purification, which usually omits fragments below 1000 bp (manufacturer's protocol). To ensure higher yield of longer fragments other methods can be used beforehand, e.g. the SR eliminator kit (PacBio, formerly “Circulomics”) or custom buffer-based precipitation [87] with enrichment options of > 10 kbp or > 50 kbp. While ultralong reads (> 50 kbp) may be produced from such DNA enriched for long fragments, this usually reduces the overall sequencing yield (which is optimal for fragments sizes of 5–15 kbp (manufacturer's guidelines). Therefore, if high coverage is desired, more moderate fragmentation to this size is required.

ONT flow cells are available in three different forms, with different sequencing output. While the classical MinION flow cells (used on the MinION and GridION platforms) have a typical output between 5 and 15 Gb, the Flongle flow cells, used with an adapter on the MinION/GridION platforms, produce less than 1 Gb, and are designed for low throughput projects (e.g. for amplicon sequencing or viral and bacterial genomes) or for test runs before starting a high coverage genome project. PromethION flow cells only run on the PromethION platform and have a capacity of 20–200 Gb. There are only minor differences in the amount of DNA library required for the three flow cell types (manufacturers protocols), so the PromethION approach is the most efficient way to generate a lot of sequence data from limited starting material.

There are several library production kits and protocols available for ONT (platform independent). The Rapid Sequencing Kit allows very fast library preparation. It involves transposase-based cleavage and adapter ligation. Therefore, it is not well suited for highly fragmented DNA and will also not produce ultra-long reads (= as it cleaves the DNA before adapter ligation). The greatest advantage is the low number of steps and speed of the protocol, enabling library production in less than 30 min. This makes it the method of choice for field sequencing and rapid results (usually not the way a genome project will be performed). The Ligation Sequencing Kit (LSK) is based on blunt-end ligation after end-repair and an optional step for “formalin-fixed, paraffin-embedded” (FFPE) repair. The protocol involves more steps and requires more time than the Rapid Sequencing Kit (2–3 h depending on experience and number of libraries processed in parallel). However, this approach delivers the highest read lengths as it corresponds to the original DNA fragment size [105, 106]. There are a couple of protocols and kits intended to maximise read lengths. A combination of the NEB Monarch HMW DNA extraction kit (New England Biolabs) and the Ultralong Sequencing Kit (Oxford Nanopore Technologies) is recommended for achieving the best read length performance (own observation).

### PacBio long-read sequencing

PacBio sequencing utilizes single-molecule real-time (SMRT) sequencing technology. In this method, a single DNA polymerase molecule is immobilized at the bottom of one of millions of zero-mode waveguides (ZMWs), which are tiny wells on a chip, with a single molecule of DNA as a template. During sequencing, fluorescently labelled nucleotides are added to the reaction. As the DNA polymerase synthesizes the complementary strand, the incorporation of each nucleotide is detected by measuring the emitted light by an optical system below the ZMWs. Each of the nucleotide bases has a corresponding fluorescent dye molecule that allows the detector to identify which base is being incorporated by the DNA polymerase during DNA synthesis. Like ONT, PacBio SMRT sequencing produces long reads, often several kilobases to tens of kilobases in length, allowing for the sequencing of complex regions of the genome, spanning repeats, and enabling the identification of structural variants.

In traditional PacBio sequencing, the raw sequencing reads have a higher error rate, compared to e.g. Illumina short-read sequencing, particularly in the form of insertions and deletions (indels) (manufacturers protocols; https://www.pacb.com/wp-content/uploads/2015/09/Perspective_UnderstandingAccuracySMRTSequencing1.pdf). The Circular Consensus Sequencing (CCS) strategy was developed to reduce these errors and increase the accuracy of the sequencing data. For this a special SMRTbell library must be created, and the CCS mode must be enabled on the instrument. The CCS method derives a consensus sequence from multiple passes of a single template molecule taken from a single ZMW, producing accurate reads from noisy individual subreads [107], High Fidelity (HiFi) long reads are then simply CCS reads with over 99% accuracy and are therefore significantly more accurate than traditional PacBio Continuous Long Reads (CLR). They have reduced indel errors and overall higher accuracy (error rate below 0.1%) [108], making them more reliable for applications such as de novo genome assembly, variant calling, haplotype phasing, and structural variant detection.

Thus, PacBio sequence reads can be described as three different groups:

1. CLR reads with a median error rate of 11% that have an insert size, the length of the DNA template between the sequencing adapter, of 25–175 kb. The CLR sequencing mode is no longer available on the new PacBio Revio instrument.
2. CCS reads require at least two full-pass subreads and have an insert size of about 10–25 kb.
3. HiFi reads areCCS reads supported by enough subreads to achieve a read quality of 0.99 or higher and have an insert size of about 10–25 kb.

Output files from Sequel systems, where on-board calling was enabled (*ccs.bam), and from Revio systems (*hifi_reads.default.bam) state the read quality with the tag “rq:f:”. The Revio systems provide HiFi reads only, whereas Sequel systems provide HiFi reads along with CCS reads with a read quality below 0.99 accuracy as well as subreads of fragments that did not create a CCS read. The latter are labelled with read quality of − 1 (rq:f:− 1).

There are two kits available: The HiFi Express Template Prep Kit 2.0 and the SMRTbell Prep Kit 3.0, the latter being the newer PacBio kit version. Both kits can be used for the PacBio Standard and PacBio Low DNA Input protocols to generate PacBio HiFi SMRTbell libraries. For the PacBio standard protocol, an input of approximately 10 µg DNA is recommended for a 3 Gb genome, but 5–6 µg DNA is often sufficient. However, both kits can also be used for PacBio's Low DNA input protocol, which requires between 300 ng and 3 ug of DNA and allows users to generate high quality genome assemblies from small organisms, e.g. from individual *Drosophila* flies [109]. It has also been possible to successfully produce libraries from only 150 ng total input DNA using this protocol. According to the provider, the genome size for this DNA input quantity is limited to 1 Gb. However, it was possible to create libraries for genomes larger than 1 Gb (own observation).

The difference between the PacBio standard protocol and the PacBio Low DNA Input protocol is mainly in the final size selection. In the standard protocol, size selection is performed with a Blue Pippin instrument (Sage Science, Beverly MA, USA), where the size selection cut is set to a higher fragment length, which usually results in higher DNA loss. The PacBio Low DNA Input protocol uses a slightly softer size selection cut, e.g. with AMPure PB beads, which is between 3 and 5 kbp. However, this results in a smaller insert size of the PacBio Low Input DNA library compared to the PacBio standard library. For samples with even less available DNA (up to 5 ng), the Ultra-Low DNA Input workflow based on amplification is available (see section Long range PCR/Whole Genome Amplification for more details).

### Improvement of assemblies by scaffolding with with additional long-range information

Initial long-read assemblies usually have more contigs than there are chromosomes. Additional sequence information might be used to generate higher-level assemblies, thus associating contigs to scaffolds [110]. The currently most prominent method, High throughput Chromosome Conformation Capture (Hi-C) is a Chromatin Conformation Capture (3 C) approach, where DNA regions of the condensed chromosomes are cross-linked and subsequently sequenced by short-read approaches. The underlying principles are that a) loci on the same chromosome are more likely to be linked; b) regions that are close to each other on the same chromosome are more likely to interact in the condensed state of the chromosome (chromatin) than regions that are more distant, although these are also linked, but to less extent. A statistical interpretation of the interaction pattern can allow the contigs to be sorted in the same order and orientation as they are arranged on the chromosome [111, 112]. It complements long-read based assemblies by providing critical spatial information that helps to reconstruct the accurate and comprehensive structure of the genome. Thus, Hi-C will improve the accuracy and completeness of genome assemblies, thereby resolving complex genomic regions to understand the higher-order organization of the genome [113–115].

Optical mapping is another, less often used way of “super scaffolding” a genome assembly [116]. This involves using enzymatic labelling of specific nucleotide sequences on ultralong (> 100 kbp) DNA molecules, which are linearized in capillaries while the labelled sequences are visualized. Labelling is based on one-stranded sequence-specific restriction enzyme cutting and nick-labelling. The cutting sites may be identified in assembly contigs and used for scaffolding based on the labelling patterns of this “optical map” [117]. Due to difficulties to generate ultralong DNA fragments and the scarcity of machines to run optical mapping it is by now less popular than chromosome conformation-capture methods (like Hi-C).

TELL seq: This method is one variant that starts with HMW DNA and generates barcode-linked short reads from long DNA fragments [118]. These short reads can be sequenced on an Illumina platform. Bioinformatically short reads will be separated according to barcodes and assembled to artificial long reads. An advantage over real long-read sequencing may be the low input required for this method, making small individuals or small tissue samples accessible to artificial long-read sequencing, as well as to phasing of haplotypes [119]. For library prep protocols and analysis pipelines (TELL-Link, TELL-Sort, TELL-Read) see https://www.universalsequences.com. Other software packages for the analysis of linked reads are available [120, 121].

### RNAseq as support for genome annotation

RNAseq data improve the annotation process by providing information about the coding parts of the genome. Even as this is just a snapshot of the genes active during the life of an organism and therefore incomplete, these data help to train the gene prediction tools to better recognise exons in this genome [122–125].

In conventional RNA-Seq, cDNA fragments for short read sequencing (100–200 bp) are analysed using computational methods to infer the original transcript isoforms [126]. This is most often sufficient for help in gene annotation of genome assemblies, where RNAseq read mappings are used as input for the annotation pipelines. However, due to the complexity of alternative splicing, many isoforms have very similar structures, and the inferred transcripts are often inaccurate [127]. If the research question needs accurate information about transcript isofoms, long-read sequencing approaches, such as ONT also provides the possibility to get full-length sequence reads for transcripts, either from cDNA or even by RNA-direct sequencing [128, 129].

Another long-read based method is PacBio Isoform Sequencing (Iso-Seq). It generates full-length cDNA sequences (up to 10 kb or more)—from 5'to the poly-A tail—without the need for cDNA fragmentation and transcript assembly and can be used for high-quality genome annotation. The PacBio Kinnex kit is basically a further development of Iso-Seq, using a method called Multiplexed Arrays Sequencing (MAS-Seq), in which smaller amplicons are concatenated into larger fragment libraries to increase throughput [130]. Requirements for the preparation of Iso-Seq or Kinnex libraries are ≥ 300 ng of high-quality total RNA input (RIN ≥ 7.0) per sample.

## Quality trimming (phase 4)

Due to the different features of ONT and PacBio long reads (variation in read length, quality, error rate) there are some differences in assembly strategies. Some assemblers can handle both types of long reads by using different parameter sets. However, we divide this part into two here, according to the two long-read sequencing platforms. There are very useful online tutorials for all aspects of analysing long-read sequence data (e.g. https://timkahlke.github.io/LongRead_tutorials).

There are standards regarding genome assembly from large consortia as VGP and DToL, which are for example implemented the pipeline pipeasm (Silva et al., 2024, biorxiv), streamlining this process. Nevertheless, we want to illuminate the various parts of a genome assembly process and its quality control, since problems during assembly are likely, especially for non-model organisms. Understanding these problems can result in higher quality of an assembly.

### ONT basecalling and quality check

Raw data in ONT sequencing is stored in pod5 (formerly fast5) data format, which has to be transformed to sequence information in fastq format. Usually this base-calling process is already performed during the sequencing run, making use of the software installed on the computer controlling the sequencing process (e.g. Guppy, Dorado). Base-calling software is constantly under development, so newer versions or other tools may perform better than the original base-calling during the sequencing run did. Therefore, it is recommended to keep base-callers up-to-date and to try a new base-calling if sequence data has been obtained some time before the assembly procedure. ONT base-callers can be run on GPUs, which reduces runtime manyfold, even with lower tier hardware. Since runtime of Guppy on GPUs is relatively short, transferring the raw data becomes the bottleneck for less comprehensive networks.

For quality control of raw data, FastQC (see Table 1 for software links) can be used for general quality checks for various types of data, but its strengths are with Illumina short reads. PycoQC and MinIONQC were developed specifically for the use with Oxford Nanopore data and both tools need access to the sequence summary files created during the sequencing run. PycoQC can deliver a quick overview about read lengths, amount and quality distribution. MinIONQC can do the same and in addition can compare the performance of various sequencing runs.

**Table 1:** Commonly used bioinformatics tools in genome projects

Tool	Github link	Bioconda package
*Mapping*		
Hisat2	https://github.com/DaehwanKimLab/hisat2	Bioconda::hisat2
bwa mem2	https://github.com/bwa-mem2/bwa-mem2	Bioconda::bwa-mem2
minimap2	https://github.com/lh3/minimap2	Bioconda::minimap2
ngmlr	https://github.com/philres/ngmlr	Bioconda::ngmlr
*Pipeline*		
pipeasm	https://github.com/itvgenomics/pipeasm	Not available
*Quality check*		
FastQC	https://github.com/s-andrews/FastQC	Bioconda::fastqc
PycoQC	https://github.com/a-slide/pycoQC	Bioconda::pycoqc
MinionQC	https://github.com/roblanf/minion_qc	Bioconda::R-minionqc
pychopper	https://github.com/epi2me-labs/pychopper	Bioconda::pychopper
porechop_ABI	https://github.com/bonsai-team/Porechop_ABI	Bioconda::porechop_abi
nanofilt	https://github.com/wdecoster/nanofilt	Bioconda::nanofilt
*HiFi Consensus*		
DeepConsensus	https://github.com/google/deepconsensus	Not available
*Assembly*		
Flye	https://github.com/mikolmogorov/Flye	Bioconda::flye
wtdbg2	https://github.com/ruanjue/wtdbg2	Not available
Canu/HiCanu	https://github.com/marbl/canu	Bioconda::canu
Hifiasm	https://github.com/chhylp123/hifiasm	Bioconda::hifiasm
Racon	https://github.com/isovic/racon	Bioconda::racon
Miniasm	https://github.com/lh3/miniasm	Bioconda::miniasm
Shasta	https://github.com/paoloshasta/shasta	Bioconda::shasta
Necat	https://github.com/xiaochuanle/NECAT	Bioconda::necat
smartdenovo	https://github.com/ruanjue/smartdenovo	Bioconda::smartdenovo
Goldrush	https://github.com/bcgsc/goldrush	Bioconda::goldrush
nextdenovo	https://github.com/Nextomics/NextDenovo	Bioconda::nextdenovo
spades	https://github.com/ablab/spades	Bioconda::spades
*Contam. check*		
FCS-GX	https://github.com/ncbi/fcs-gx	Bioconda::ncbi-fcs-gx
blobtools	https://github.com/DRL/blobtools	Bioconda::blobtools
blobtoolkit	https://github.com/blobtoolkit/blobtoolkit	Bioconda::blobtoolkit
markerscan	https://github.com/CobiontID/MarkerScan	Not available
*Scaffolding*		
SSPACE- LongRead	https://github.com/Runsheng/sspace_longread	Not available
SSPACE	https://github.com/nsoranzo/sspace_basic	Bioconda::sspace_basic
SLR	https://github.com/luojunwei/SLR	Etetoolkit::slr
ARCS	https://github.com/bcgsc/arcs	Bioconda::arcs
LRNA- scaffolder	https://github.com/CAFS-bioinformatics/L_RNA_scaffolder	Not available
qc3 C	https://github.com/cerebis/qc3C	Bioconda::qc3c
Picard	https://github.com/broadinstitute/picard	Bioconda::picard
chromap	https://github.com/haowenz/chromap	Bioconda::chromap
YaHS	https://github.com/c-zhou/yahs	Bioconda::yahs
*Telomeres*		
tidk	https://github.com/tolkit/telomeric-identifier	Bioconda::tidk
*HiC visual*		
Juicer	https://github.com/aidenlab/juicer	Bioconda::juicer
Rapid curation	https://gitlab.com/wtsi-grit/rapid-curation	Not available
*Assembly quality*		
quast/quast-LG	https://github.com/ablab/quast	Bioconda::quast
Busco	https://gitlab.com/ezlab/busco	Bioconda::busco
Compleasm	https://github.com/huangnengCSU/compleasm	Bioconda::compleasm
Meryl	https://github.com/marbl/meryl	Bioconda::meryl
Merqury	https://github.com/marbl/merqury	Bioconda::merqury
Bamqc	https://github.com/s-andrews/BamQC	Bioconda::qualimap
*Haplopurging*		
Redundans	https://github.com/Gabaldonlab/redundans	Bioconda::redundans
purge_haplotigs	https://github.com/skingan/purge_haplotigs_multiBAM	Bioconda::purge_haplotigs
purge_dups	https://github.com/dfguan/purge_dups	Bioconda::purge_dups
HapSolo	https://github.com/esolares/HapSolo	Biocomda::hapsolo
*Repeats*		
Repeatmasker	https://github.com/Dfam-consortium/RepeatMasker	Bioconda::repeatmasker
Repeatmodeler	https://github.com/Dfam-consortium/RepeatModeler	Bioconda::repeatmodeler
*Annotation*		
Augustus	https://github.com/Gaius-Augustus/Augustus	Bioconda::augustus
Braker 3	https://github.com/Gaius-Augustus/BRAKER	Bioconda::braker3
funannotate	https://github.com/nextgenusfs/funannotate	Bioconda::funannotate
helixer	https://github.com/weberlab-hhu/Helixer	Not available
Toga	https://github.com/hillerlab/TOGA	Not available
interproscan	https://github.com/ebi-pf-team/interproscan	Bioconda::interproscan
EggNOGmapper	https://github.com/eggnogdb/eggnog-mapper	Bioconda::eggnog-mapper
Fantasia	https://github.com/MetazoaPhylogenomicsLab/FANTASIA	Not available

Read filtering, quality trimming and adapter removal can be done with pychopper and porechop_ABI. Nanofilt can perform additional filtering steps, e.g. excluding reads that are smaller than a given length. In general, the quality scores of ONT data are not as good as those of short read approaches, so that harsh filtering will omit much of the sequencing data.

### PacBio HiFi base-calling, preprocessing and quality check

When sequencing with PacBio in CCS mode on the Sequel I/II/IIe systems, there are two ways to obtain the HiFi reads. Either so-called on-board calling is switched on to generate HiFi reads directly on the PacBio machine or HiFi calling is performed from the subreads afterwards. The advantage of on-board calling is that a much smaller amount of data needs to be transferred from the sequencing machine or a sequencing provider and the computation regarding HiFi calling is already done. The disadvantage is that a) the subreads are lost during this process and one cannot redo the HiFi calling and b) on-board calling on the Sequel systems is done with tools developed by PacBio (*ccs*), which are not as good as alternative tools such as DeepConsensus [131]. The pipeline around DeepConsensus is computationally more demanding but typically yields 10% more data per SMRT cell. Especially for projects with limited financial resources, HiFi calling with DeepConsensus can be advantageous to get more data for the same price. For the newer Revio system, on-board HiFi calling is performed using the DeepConsensus pipeline and cannot be disabled.

Briefly, the pipeline to run DeepConsensus consists of first running PacBio’s *ccs*, to get all CCS reads—including CCS reads with quality below HiFi level and second running PacBio’s *actc* to align the subreads against the previously created CCS reads. Finally, DeepConsensus processes this alignment, and the CCS reads to create the final HiFi reads.

To complete the HiFi calling in a reasonable timeframe, it is recommended to process the subreads of one SMRT cell in chunks. Since *ccs* and *actc* have this functionality already implemented, the only work is to adapt these processes to the available commute system. For example, when splitting the data of one SMRT cell into a thousand chunks, each chunk is typically small enough to be processed on 4 threads and 25 Gb of RAM in two to three hours depending on the amount of subreads present as output. In practice, HPC job scheduling systems, e.g. slurm [132] can easily handle thousands of jobs in a job array, where each array element corresponds to one chunk of sequence. To minimize disk space use, we recommend removing temporary files for each chunk, once it has been successfully completed. Once all the array jobs have been completed, one should carefully check that they all finished without errors – for example due to time or memory limits. If there are no errors, the one thousand fastq files can simply be concatenated.

Before running the assembly, it is useful to count the total length of all available HiFi reads and evaluate their length distribution (e.g. calculate average length or N50, see section assembly contiguity for explanation). This will give you an idea of whether an assembly might be worth trying or not. By dividing the total HiFi Gb by the estimated genome size a theoretical coverage can be estimated. Depending on the research question, coverages as low as 4× may be sufficient e.g. for a complete mitochondrial genome and some contigs containing nuclear genes. For reference quality assemblies a coverage around 30× is adequate but depending on the complexity of the genome, good results can already be achieved with approximately 20×, (e.g. [133]). The HiFi N50 can be used to estimate the level of fragmentation regarding the contig level assembly. Shorter HiFi reads will less likely bridge repeats, which will lead to a more fragmented assembly and collapsed repeats.

## Genome assembly (phase 5)

Although there are many assemblers which are able to process PacBio HiFi data, such as Flye [134], wtdbg2/redbean [135] and HiCanu [136], hifiasm [79, 137] usually performs best in terms of speed, contiguity and accuracy. If needed, the assembly can be phased in this stage, when using hifiasm, especially when including also information from Hi-C.

There are a number of long-read assembly tools for error-prone reads such as older ONT data, including Canu [138], Racon [81], Minimap2/Miniasm [139, 140], Flye [134], Shasta [141], wtdbg2 [135], NECAT [142], smartdenovo [143], GoldRush [144], Nextdenovo [145], and spades [146]. Spades was written for short-read data but can include long-reads as supporting data. Although Canu is still one of the most accurate long-read assemblers, the requirements of RAM and disc space make it difficult to work on larger genome datasets with limited computing resources. The other tools are more lightweight, with medium or low computational requirements and varying levels of accuracy (see chapter on resources). Benchmarking studies with bacterial genomes [147, 148] show the pros and cons for a couple of tools. However, bigger eukaryotic genomes with complex repeat landscapes provide more challenges than bacterial genomes. Benchmarking here shows Flye to be among the best performing assemblers for ONT data [149], however hifiasm since version 0.21 as well can deal with ONT data (R10) and performs quite well (own observation).

Usually, assemblies can be set up from 20× coverage sequence data, but the contiguity and completeness of a genome is much better at 30–50 × coverage or more. Canu by default selects the longest reads that sum up to 20× coverage for the initial assembly steps. Canu, Flye, and wtdbg2 include steps for correcting sequencing errors due to read overlaps, while minimap/miniasm does not. Assemblies from ONT flow cells prior to v.10 will include non-random errors (most often a few bp are skipped in a non-random way) that need to be corrected with other sources of sequence data, e.g. low or medium coverage (10 − 20x) short read data, at best from the same specimen. This “polishing” can be conducted with Pilon [150], for example, which corrects long-read assemblies with mapped short-reads. On the downside, correction with short reads can have an impact on repeat elements in the assembly, leading to an “overcorrection” of these due to the mapping of a multitude of repeat reads to all copies of the repeat. New ONT flow cells (generation R10 and higher) have a much lower error rate and errors are random [101], so there is no need for correction of ONT based assemblies anymore.

### Contamination check

Contamination problems arise in cases where other organisms are present in the sample: in many species intracellular parasites and symbionts will be sequenced alongside the target species [151, 152]. A similar problem is faced with gut content, e.g. when whole specimens were used for DNA extraction. It might also be difficult to extract DNA from endoparasite target species without contamination from their host; here often free larval stages or eggs have to be used [153]. Especially when dealing with small individuals and/or small amounts of DNA, the ratio between target and contamination may be biased. While DNA contamination coming from the addition of sequencing adapters could be identified easily during quality checks of sequencing reads before starting the assembly process, identifying natural contaminations in the sequencing reads would be computationally very demanding and is thus usually done after the assembly is finished. A prominent example for misinterpreting contaminations as horizontal gene transfer was presented with the genome of the tardigrade *Hypsibius dujardini* [154–156]. As well, contaminations may lead to errors in the annotation process, by annotating genes that do not belong to this organism [157].

NCBI Foreign contamination screen relies on sequence similarity only but can be automatized easily. With FCS-adaptor and FCS-GX [158] artificial and biological contamination respectively, can be identified and removed. By providing the taxonomic identifier (NCBI taxid) of the target organism, FCS can distinguish parts of the assembly that most likely originate from the target and those which are not. The downsides of this method are a) the incomplete GX database, which may miss contamination that is yet not represented fully in the database and b) biological contamination from species too closely related to the target species may be missed.

**Fig. 2:**
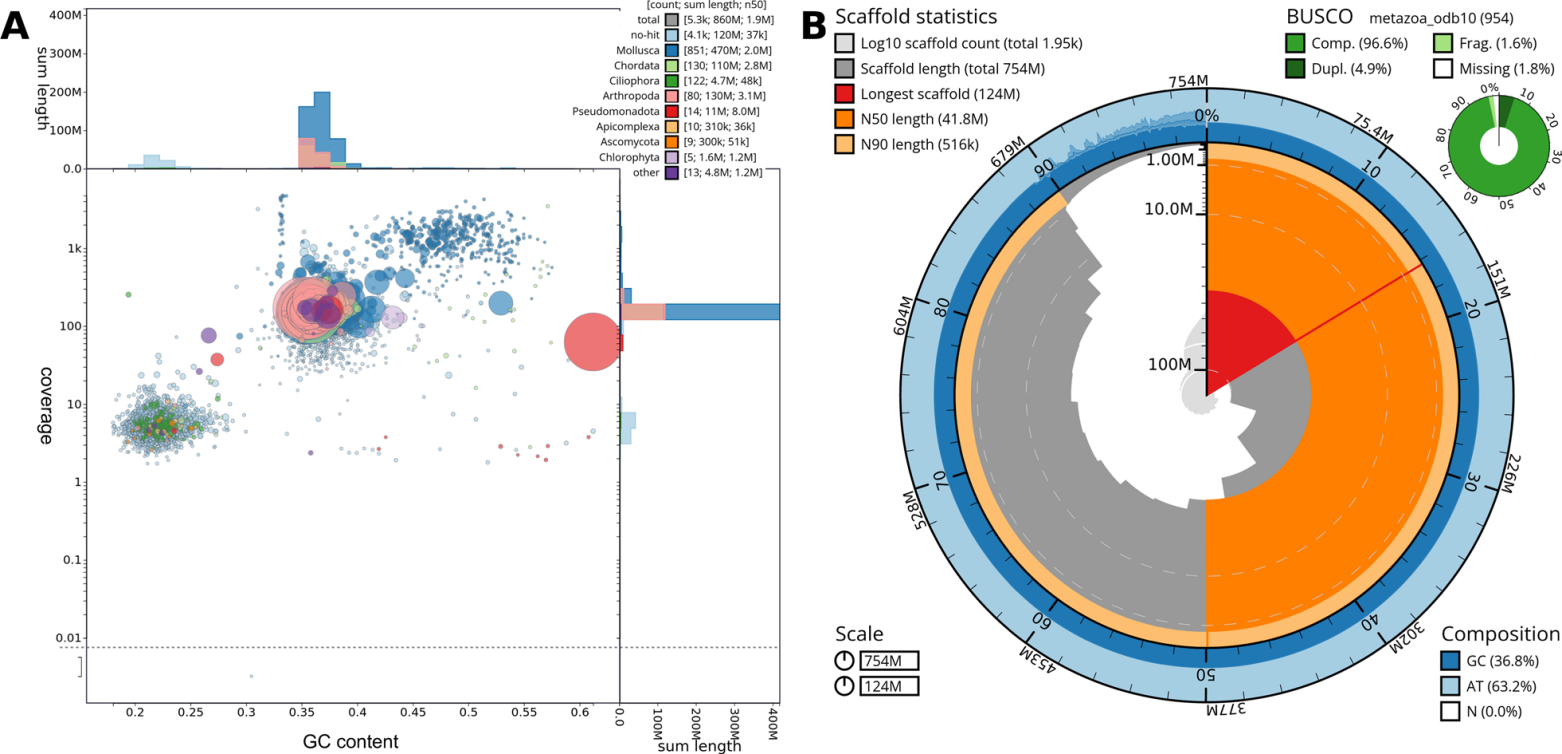
Assembly quality assessment with blobtoolkit. A) Blobplot of FCS filtered contigs: Each circle represents a sequence of the assembly. Size and color of the circle correspond to the size and taxonomic assignment of the respective sequence. Note that the cluster of contamination on the bottom left contains sequences assigned to Ciliophora and no-hits (amongst others), which were not filtered out by FCS. B) Snailplot of chromosome scale scaffolds: Graphical representation of various contiguity statistics of a genome assembly. Main plot (center): Clock wise the absolute and relative length of the assembly is displayed. The outer light and dark blue ring show GC content at a respective position of the assembly. Dark grey columns in the middle show the number of the sequence and its length with height and angle, respectively. Major contiguity statistics as longest sequence, N50 and N90 are highlighted in red and orange tones. Additionally, on the top right a graphical representation of BUSCO results is shown (Figure reproduced from Männer et al. 2024)

Another widely used method, blobtools [159] or the more inclusive blobtoolkit [160], involve clustering contigs and/or scaffolds regarding read coverage and GC content. Further information is added by taxonomic classification, which is done according to the taxon-wise sum of blast scores per sequence. By default the taxonomic assignments are at the phylum level to easily distinguish between bacterial, fungal, animal and plant contaminations. If necessary taxonomic assignments can be done with lower taxonomic levels, allowing for a more detailed analysis. There are two basic assumptions of this method. First, contamination has a different GC content as the target species. This is particularly true when dealing with, for example, metazoan genomes as targets and bacteria as contaminants which generally differ in GC content (e.g. *Wolbachia* symbionts have a low GC content of about 35% versus 40–50% in Metazoa) [161]. Second, it is assumed that contamination should be relatively under- or overrepresented in the DNA sample, the sequencing library and the resulting data. For example, for metazoan parasites of the target organism, fewer reads will be sequenced from such sources, resulting in a lower coverage of contigs assembled from these reads. Otherwise, bacterial symbionts may be present in high abundance and their genomes might be sequenced with a higher coverage than the target genome. An example of such an GC vs. coverage plot from the sea slug *Elysia timida* [91] generated with blobtoolkit can be seen in Fig. 2A. Here, e.g. the cluster containing dark green circles on the bottom left corresponds to contigs assigned by blobtoolskit to Ciliophora. GC content and coverage clearly separate the clusters of contigs representing the target species genome and this contamination. Manual curation was performed to prevent filtering false positive hits.

In general we recommend running FCS first, but subsequently checking for further contamination with a blobplot. Regarding only the coverage of a contig or scaffold in an assembly, lower coverages can be caused by several other reasons. For example, haplotigs, contigs of the same genomic locus that are represented more than once (e.g. twice for diploid species) in an assembly due to heterozygosity, will have a fraction of the expected coverage (e.g. half for diploid species). Other problems associated with lower-than-expected target coverage may be due to incomplete representation of the genome in the DNA extraction or library of the sample, or problems with amplification or sequencing of certain regions (e.g. if a genome amplification was done prior to sequencing). The blobplot becomes more difficult to interpret, when sequences of an assembly are distributed along a continuum of GC content and/or coverage, as well as when contigs are rather short, as in assemblies from low coverage data or pure short-read assemblies [160]. Furthermore, taxonomic assignment by BLAST search can be misleading, when species which are underrepresented in the database are used for sequence similarity searches [162]. In these cases, matches to the closest sequence in the database may not reflect the true origin but rather a more distant evolutionary relationship. For example, conserved genes or domains can be represented by other taxa than the target. NCBI’s nucleotide database (nt) represents mammals, vertebrates and partially insects very well but when it comes to molluscs or other non-insect invertebrates, taxonomic assignment should be treated with caution, due to the false positive hits. We strongly recommend additional manual curation of the contigs suggested to be contaminated. There are also tools that allow for the co-assembly of genomes from symbionts and parasites such as markerscan (https://github.com/CobiontID/MarkerScan).

### Scaffolding with Hi-C data

In the context of genome assembly, scaffolding describes the process of determining the order and orientation of sequences (e.g. contigs). While scaffolding with long reads such as PacBio CLR (e.g. SSPACE-LongRead [163]) and/or ONT reads (e.g. SLR [164]) as well as mate-pair reads (e.g. SSPACE [165]), linked reads (e.g. ARCS [166]) or even transcripts (e.g. L_RNA_scaffolder [167]) has been used to overcome limitations of short-read based assemblies, as these techniques usually do not reach chromosome level standard. With the replacement of Dovetail Hi-C by Omni-C technology and the introduction of Arima Hi-C, powerful tools are available to generate chromosome level assemblies.

For most projects it may be possible to obtain enough high molecular weight DNA from a single specimen for PacBio and Hi-C sequencing. However, this may not be possible, especially for species with very small individuals. While pooling of individuals for HiFi sequencing is not recommended, this is less of an issue when generating Hi-C data. The difference is that e.g. PacBio HiFi reads are used directly to determine the sequence (contigs), whereas Hi-C data are used to determine the order and orientation of the contigs. Sequences from the Hi-C data are not incorporated into the contigs but are only used as anchors by mapping the Hi-C reads to the contigs. This means that sequencing a different individual or even a pool of specimen is a reasonable option here, if the mapping is still possible without bias (e.g. due to an excess of SNP-sites) and the karyotype is identical.

As Hi-C library preparation is a challenging process, results may vary. Therefore, it may be useful to test the success of a Hi-C library preparation before proceeding with deep sequencing or extensive analyses. The tool qc3C [168] can produce quality checks of Arima or phase Hi-C data for comprehensive insights on cross linking success. These quality checks can be performed without a reference (k-mer based) or with a reference (mapping based). In order to scaffold contigs, Hi-C reads need to be mapped against them. The official pipeline from Arima (https://github.com/ArimaGenomics/mapping_pipeline) is based on bwa mem [169] and samtools [170] for mapping the reads as well as Picard (https://github.com/broadinstitute/picard) and additional scripts from the pipeline to filter and combine the mappings. Alternatively, chromap [171] can be used to map Hi-C reads, which is faster than bwa mem and all filtering steps are already included in this tool without relying on installing further software packages. Once the mapping is complete, a tool for scaffolding needs to be applied. Currently YaHS [172] appears to be one of the fastest and most accurate options for this task. Next to using the Hi-C signal to join contigs into scaffolds, these tools usually include options to correct mis-assemblies e.g. by breaking contigs.

Visualization of Hi-C data is done based on so-called contact maps. These maps depict in a two-dimensional way, where Hi-C read pairs support the current order and orientation. In a contact map, the genomic sequence should be imagined on the diagonal. Both remaining triangles are mirrored and contain the same information. Each coordinate in the contact map can be assigned to two locations in the assembly, which represent locations of both reads from a pair. That means coordinates close to the diagonal represent pairs with small and coordinates far from the diagonal pairs with larger insert size. Changes in colour and/or contrast show how many read pairs support linkage of respective loci. Orientation may be easier to determine for larger contigs and/or scaffolds, as there may be a gradual signal.

Sometimes the Hi-C signal between e.g. chromosome arms is not clear enough to orient them without doubt. In general, but especially in those cases searching and depicting abundance of telomeric repeats is very helpful to determine the correct orientation. Finding telomeric repeats can be done with tidk [173] (tidk search –string ACCCTA –extension bedgraph) for example.

With the rise of phased assemblies, so-called dual curation was introduced. In dual curation, the contact maps of both haplotypes are merged and represented in quadrant two and four respectively. The correspondence between the haplotypes is displayed in quadrants one and three (again mirrored with identical information). The advantage of dual curation is the ability to spot parts, which were wrongly assigned to one of the haplotypes. The process of inspecting and correcting these contact maps is called manual curation.

A comprehensive guide on interpreting contact maps can be found in the documentation of the rapid curation pipeline (https://gitlab.com/wtsi-grit/rapid-curation/-/blob/main/Interpreting_Hi-C_Maps_guide.pdf).

In practice visualization is done in either Juicebox from the Juicer package [174] or PretextView from rapid curation (https://gitlab.com/wtsi-grit/rapid-curation). Although Juicebox is older and the graphical user interface is not very intuitive, YaHS is still compatible. The disadvantage is that Juicebox is not suitable for dual curation, since the output of e.g. YaHS needs to be converted into a Hi-C file, which is used as input for Juicebox. For PretextView any mapping of Hi-C reads can be converted via PretextMap into a pretext file, which is in turn used as input.

### Assembly contiguity

The most widely used metric to describe the quality of an assembly is the contiguity, i.e. how many different contigs are present and how is their length distribution. Ideally, there are few sequences that are long (chromosome length). Mean sequence length does not reflect the quality in a meaningful way, as sequence lengths are usually not uniformly distributed within an assembly, with few long sequences and many short sequences reflecting repeats that are difficult to place by the assembly tools. Here, mean sequence length will be low, ignoring the few long sequences. In turn the commonly used N50 value represents the length of the sequence, where 50% of the assembly’s total length is in sequences of this length or longer after the sequences have been sorted by length [175]. With evenly distributed contig lengths mean and N50 values are close to each other. If contig lengths are unevenly distributed, the few long sequences contribute more to the N50 value, which will be much higher than the mean sequence length. Analogous, e.g. N75, N90 and N99 can be calculated, showing the length at 75, 90 and 99% of the assembly’s total length, respectively. The disadvantage of the N50 is that values calculated from assemblies of different lengths are not directly comparable. Metrics such as the NG50 are more appropriate because they use the estimated genome size rather than the length of the assembly (which might be inaccurate due to missing parts or collapsed repeat regions), making it reasonable to compare assemblies from species with similar genome sizes. To calculate e.g. the NG50, the total length of the assembly needs to reach at least 50% of the estimated genome size. As for the N50, basically any percentage of the genome size estimate can be applied. There are other methods that reflect contiguity, such as the L50 and LG50, which indicate how many sequences are needed to reach 50% of the assembly’s total length and estimated genome size, respectively [176]. Often L95 or even L99 values are used to show how close this metric is to the karyotype and how little of the assembly’s total length is not linked to larger, chromosome scale scaffolds. Useful tools to generate these metrics are QUAST/QUAST-LG [176, 177]. These tools also have sophisticated additional features that can compare assemblies with reference genomes. With more and more almost complete chromosome-level assemblies, N50/L50 metrics become meaningless for comparisons, so other metrics such as number of contigs/chromosome number or placed vs. unplaced contigs may be useful values, see a discussion in [178].

### Assembly completeness

A meaningful and easily achievable metric to show the quality of an assembly is to compare its total length to the estimated genome size. The closer the total length is to the estimated genome size the better. Assembly lengths less than the estimated genome size can be consequence of difficult to assemble parts like telomeres but may also indicate problems such collapsed repeat regions. An assembly size bigger than expected may hint to many haplotypic duplications [20], which may be identified by comparing the coverage between contigs.

Completeness of a genome assembly can be tested by trying to place all the raw sequence data on it. When dealing with accurate reads such as Illumina or PacBio HiFi it is useful to compare the k-mers found in the reads with the k-mers found in the assembly. First the completeness of these k-mers can be calculated, which should be close to 100% if all reads are assembled. Second, an error rate of the assembly can be calculated, by assuming that k-mers found only once in the assembly are base errors [179]. The consensus quality value (QV) is a logarithmic representation of the error rate, with higher values corresponding to higher accuracy (e.g. Q30 refers to an accuracy of ~ 99.9%, Q40 to ~ 99.99%). Both, k-mer completeness and QV, can be calculated using Meryl (for the k-mer counting) and Merqury [180]. In addition to these values Merqury provides informative plots on k-mer multiplicity, giving information on e.g. k-mer coverage and heterozygosity.

Next to k-mer based analyses, mapping based quality checks can reveal problems of an assembly. To do so, the reads used for assembly will be mapped to the assembly itself. For PacBio HiFi reads and ONT reads mappers with presets for respective technologies are recommended, e.g. minimap2 [139, 140]. A summary report can subsequently be generated using Qualimap bamqc [186], which provides a variety of informative plots and statistics. Firstly, the proportion of mapped reads should be high, to ensure that most of the reads are represented in the assembly. This value can be roughly compared with Merqury’s k-mer completeness. If a larger fraction is not mapped, this could indicate contaminations, which are not well covered to end up in the assembled contigs. Second, the shape of the coverage distribution can show several points. Ideally, the theoretical coverage (total sequenced base pairs divided by the estimated genome size) matches the modal value of the mapping coverage. Furthermore, the coverage is evenly distributed around the modal value. A bimodal distribution usually indicates a larger fraction of haplotigs (seperate contigs for each homologous part of a diploid chromosome), which have more positions with only half of the expected coverage. For chromosome level assemblies, sex chromosomes with half the coverage of the autosomes may be visible, if they are large enough. A coverage distribution with a long tail of high coverage could indicate many loci of collapsed repeats in the assembly [187].

The recovery of bench-marking universal single-copy orthologs (BUSCO) [181, 182] is one of the most widely used metrics to assess the quality of an assembly. Currently BUSCO provides orthologous gene sets for nearly two hundred taxonomic groups. These sets are built upon species with genome annotations available of this taxonomic group. Even as only a subset of all genes (= the single-copy orthologs) from a species are considered, it is assumed that the recovery of BUSCOs can be extrapolated to the entire gene set from the species of interest. A BUSCO analysis returns whether a searched gene is found in the assembly under study complete and single copy, complete and duplicated, fragmented or if the gene is missing. If a certain percentage of the searched set is found complete and in single copy in a given assembly, one can assume that approximately the same percentage of all expected genes are present in the assembly [183]. It is important to keep in mind how many and which species contributed to a particular set. For example, the mollusca_odb10 set contains more than 5000 genes from only seven different species (one from Cephalopoda, three from Bivalvia and three from Gastropoda). Given the extreme diversity of Molluscs, this set is not very representative to evaluate genome assemblies from more distantly related species than those included in the set. In such cases using a more general set, e.g. Metazoa instead of Mollusca, may give more meaningful results, although fewer genes are being searched. As the assembly is screened for single copy orthologs, the number of duplicated BUSCOs should be low. Higher fractions of duplicated BUSCOs may indicate assembly errors such as the presence of haplotypic duplications in the assembly or biological differences from the applied set, e.g. duplication events in the genome under study. The percentages of fragmented and missing BUSCOs should be as low as possible, as higher fractions may indicate for example high levels of fragmentation of the genome assembly or an unusually high error rate. On the other hand, biological differences could also explain deviations. For example, genes in the genome under study may differ too much to be found with the BUSCO model for that taxonomic level. Besides quality assessment, the BUSCO approach can also be used in helping with gene annotation or to set up phylogenomic datasets [184].

Recently, a reimplementation of BUSCO, compleasm [185], was published, which uses the same sets as BUSCO but a more effective protein-to-genome comparison approach. The main practical differences are the lower runtime and higher accuracy compared to BUSCO. Additionally, compleasm distinguishes between fragmented genes, which are only partially found (F) and fragmented genes, which parts are found on completely different contigs of the assembly (I). As the sensitivity to more distant homologs is lower in compleasm than in BUSCO, it is suggested to compare the results of compleasm and BUSCO.

Current publications describing genome assemblies often provide a snail plot created with BlobToolKit to show contiguity and BUSCO completeness in one figure [160]. The example displayed in Fig. 2B was taken from [91]. The scaffold lengths distribution is shown in dark grey, by scaling the plot radius to the longest scaffold in the assembly (shown in red). The logarithmically scaled cumulative scaffold count (1.95 k) is presented in the centre of the plot in light grey. The dark and light orange shaded arcs show N50 (41.8 Mb) and N90 (516 kb) lengths, respectively.

### Haplotig purging and phased assemblies

Given sufficient read coverage and substantial heterozygosity, assembly tools tend to deliver parts of the assembly as haplotigs (haploid contigs). A high number of duplicated genes in the BUSCO analysis would be evidence of this. Similarly, analysis of the coverage of contigs/scaffolds may give some indication of the extent of haploid contigs (but these may also be part of the sex chromosomes in the heterogametic sex). A mix of haploid and diploid contigs is misleading annotation and subsequent analysis steps. Haplotigs can be purged or fused by using specialized tools, such as redundans [188], purge_haplotigs [189], purge_dups [190], or HapSolo [191]. On the other hand more and more approaches desire phased assemblies, where both homologous chromosome sets are part of the assembly [192, 193].

## Annotation of repeats and protein-coding genes (phase 6)

The main focus of this review has been on sequencing and assembly of de-novo genome projects. Therefore, this chapter only gives a brief overview of the next steps—the annotation of the different functional elements of a genome.

Structural annotation involves the identification of repeat elements and protein coding regions. Transposable elements (TE) often contain open reading frames, which would interfere with most protein prediction pipelines (especially problematic when a TE is present in an intron). Therefore, the first step in annotation is the identification of TEs and other repeat elements (e.g. simple repeats) and their masking prior to the protein annotation step [194, 195].

First choice here is still Repeatmasker/Repeatmodeler. Some repeat information is available for model organisms, e.g. in the DFAM database [196]. Here RepeatMasker (https://www.repeatmasker.org) can be used directly with the respective repeat libraries. For other organisms repeat libraries have to be constructed from the assembly information. RepeatModeler [197] is a commonly used tool for this task, actually a pipeline that combines several repeat identification tools. In addition, there are several tools that are specialized on particular repeat families and give additional evidence.

RepeatModeler (alone or in combination with other tools) will produce a lot of redundant information as well as false positives (rRNA genes and some highly similar protein coding gene families may also be identified). There are some good reviews giving advice on how to make thorough repeat annotations manually and/or semi-automatically from the initial RepeatModeler output [198–200]. Many more specialised tools exist for specific repeat families, for a broad overview on methods, protocols and tutorials see also TE-hub (https://tehub.org).

Methods for structural annotation are generally divided into ab initio or evidence-based approaches. Hidden Markov Models (HMMs) can be created and trained to annotate genes from a particular species and to recognize intron/exon boundaries without additional data such as RNA-seq or Iso-Seq. A primer on HMMs can be found in [201]. Annotation pipelines typically include both model-based and evidence-based methods for more precise detection of gene boundaries. In a best-case scenario, there is evidence for transcription from RNAseq data available, as well as a model, which supports or even extends the evidence-based annotation.

Annotation of protein-coding genes [202] can be done with Augustus [203], which is part of well-known annotation pipelines such as Maker [122] and Braker [124, 125]. Funannotate (github.orf/nextgenusfs/funannotate) is another alternative, originally intended to be used for fungal genomes, but now also well adapted for many other eukaryotic genomes. There are also promising machine-learning approaches for the structural annotation of genes [204–206]. Tools for comparative annotation can also help, if related species already have a genome annotation, e.g. the comparative annotation toolkit [207], and TOGA [208].

Structural annotation is usually followed by functional annotation, which assigns certain characteristics to a protein sequence. For instance, Gene Ontology (GO) terms [209, 210], in which metabolic pathways the protein is likely to be involved in, e.g. using the Kyoto Encyclopaedia of Genes and Genomes (KEGG) [211–213], Superfamily [214] and many other features such as domains, being transmembrane or general similarity. Functional annotations can be performed using tools such as InterProScan [215], which combines many databases and tools, eggNOG-mapper [216] or Fantasia [206].

For non-model organisms, structural and functional annotation can be difficult due to insufficient evidence and underrepresentation in databases. Furthermore, fragmented assemblies will lead to more fragmented annotations (e.g. one gene split into two on two different contigs). Shorter and fragmented protein sequences in structural annotations are subsequently more difficult to be assigned to functions.

## Making datasets accessible for the public

In order to make the generated data and results usable for the scientific community, authors need to act according to the FAIR principle (Findability, Accessibility, Interoperability, and Reuse of digital assets) [217] by at least uploading the raw sequencing data, the assembly and annotation to one of the members of International Nucleotide Sequence Database Collaboration (INSDC), namely a) The Research Organization of Information and Systems and National Institute of Genetics (RIOS-NIG; https://www.ddbj.nig.ac.jp/), b) The European Molecular Biology Laboratory and European Bioinformatics Institute (EMBL-EBI; https://www.ebi.ac.uk/ena) and c) The National Library of Medicine and National Center for Biotechnology Information at the National Institutes of Health (NLM-NCBI; https://www.ncbi.nlm.nih.gov/). To ensure reproducibility and describe the assembly process as well as downstream analyses, a peer reviewed publication is additionally recommended, including access to protocols of wet lab procedures and bioinformatic analyses.

## Conclusions and outlook

Despite modern sequencing methods becoming more accurate and are now able to sequence longer fragments, it is still not possible to determine the exact sequence of a whole chromosome by reading it completely. Therefore, sequenced bases must provide a multiple of coverage of the genome size and de novo genome assembly steps are needed to provide a genome reference sequence. However, current sequencing methods enable us to unlock information more easily than ever before. More and more accurate real-time single-molecular sequencing techniques, combined with higher-level scaffolding, allow for chromosome-scale assemblies, with phasing and the detection of epigenetic modifications as well as structural variants like copy-number variations or large inversions [18]. Genome data can now be generated in small laboratories with limited budgets even for non-model organisms, while world-wide genome initiatives aim for providing genomic data of the highest possible quality for many more organisms. It will remain a challenge to do comparative genomics with genomes of varying quality by means of assembly and annotation. We can expect that due to the large genome initiatives there will be better standards for genome assembly and annotation. To stay updated with current standards researchers may refer to the latest standards of these initiatives like e.g. Earth Biogenome [23] or Darwin Tree of Life [22]. We can also expect that there is more hidden genomic variation inside species boundaries detected with more accurate genome assemblies.

While the assembly process might be more streamlined, and even automated soon, reliable annotation of genomes seems to be more difficult to achieve. To discover the biological meaning of a genome, structural and functional annotations are needed. Annotation pipelines for repeats and proteins still vary enormously in output, making it difficult to compare results between different laboratories and different species. This is a field where progress is to be expected soon from machine learning approaches, as well as in uncovering the functions of the “dark proteome” [206]. Also, comparative annotations could be performed more reasonably, as the taxon coverage is now providing more and more genomes from closely related species. Population genomics and comparative genomics will continue to unravel the molecular basis of evolutionary processes. It is of major importance to make assemblies and annotations reproducible and provide availability of all analysis parameters and scripts [218], as well as to provide open access to sequence information, annotations and protocols of laboratory procedures. General rules for structural and functional annotations would make it easier to compare genomes of different organisms analysed in different labs or initiatives. As medical science approaches are often a forerunner for general biology fields, we can easily predict that comparative genomics will in future focus much more on the influence of structural variation, copy-number variations, non-coding elements, and repeat elements on the evolution of animals, plants and other organisms.

## Data Availability

Not applicable (review article).

## Abbreviations

bp: Base pairs
BUSCO: Bench-marking universal single-copy orthologs
CCS: Circular consensus sequence
CLR: Continuous long-reads
DNA: Desoxyribonucleic acid
DOP: Degenerate oligonucleotide
FFPE: Formalin-fixed paraffin-embedded
Gbp: Gigabasepairs
Hi-C: High throughput chromosome conformation capture
HiFi: High fidelity
HMW: High molecular weight
HPC: High performance computing
LIANTI: Linear amplification via transposon insertion
MDA: Multiple displacement amplification
ONT: Oxford nanopore technology
PacBio: Pacific biosciences
PCR: Polymerase chain reaction
PEP: Primer extension pre-amplification
RNAseq: Sequencing of RNA transcripts
SMRT: Single-molecule real-time
SNP: Single nucleotide polymorphism
SRE: Short read eliminator
TE: Transposable element
WGA: Whole genome amplification
ZMW: Zero-mode waveguides
